# Cellular and Molecular Events Leading to Paraquat-Induced Apoptosis: Mechanistic Insights into Parkinson’s Disease Pathophysiology

**DOI:** 10.1007/s12035-022-02799-2

**Published:** 2022-03-19

**Authors:** Wesley Zhi Chung See, Rakesh Naidu, Kim San Tang

**Affiliations:** 1 Jeffrey Cheah School of Medicine and Health Science, Monash University Malaysia, 47500 Bandar Sunway, Selangor Malaysia; 2 School of Pharmacy, Monash University Malaysia, 47500 Bandar Sunway, Selangor Malaysia

**Keywords:** Alpha-synuclein, Apoptosis, Brain-derived neurotrophic factor, Endoplasmic reticulum stress, Nitrosative stress, Oxidative stress, Paraquat, Parkinson disease

## Abstract

Parkinson’s disease (PD) is a progressive neurodegenerative disorder characterized by the cardinal features of tremor, bradykinesia, rigidity, and postural instability, in addition to other non-motor symptoms. Pathologically, PD is attributed to the loss of dopaminergic neurons in the substantia nigra pars compacta, with the hallmark of the presence of intracellular protein aggregates of α-synuclein in the form of Lewy bodies. The pathogenesis of PD is still yet to be fully elucidated due to the multifactorial nature of the disease. However, a myriad of studies has indicated several intracellular events in triggering apoptotic neuronal cell death in PD. These include oxidative stress, mitochondria dysfunction, endoplasmic reticulum stress, alteration in dopamine catabolism, inactivation of tyrosine hydroxylase, and decreased levels of neurotrophic factors. Laboratory studies using the herbicide paraquat in different in vitro and in vivo models have demonstrated the induction of many PD pathological features. The selective neurotoxicity induced by paraquat has brought a new dawn in our perspectives about the pathophysiology of PD. Epidemiological data have suggested an increased risk of developing PD in the human population exposed to paraquat for a long term. This model has opened new frontiers in the quest for new therapeutic targets for PD. The purpose of this review is to synthesize the relationship between the exposure of paraquat and the pathogenesis of PD in in vitro and in vivo models.

## Introduction

PD is the second most common and progressive neurodegenerative disease after Alzheimer’s disease, affecting approximately six million people over the age of 60, worldwide [1]. The cardinal features of PD include tremor, bradykinesia, rigor, and postural instability [2]. These motor symptoms are due to the selective degeneration of the dopaminergic neuronal cells located in the substantia nigra pars compacta (SNpc), in addition to increased dopamine deficit in the striatal axonal projection area. However, PD is also often associated with other non-motor symptoms that commonly manifest in the gastrointestinal tract, such as gastric reflux, constipation, and swallowing difficulties, in addition to cognitive impairment and neuropsychiatric symptoms, such as depression, anxiety, sleep behaviors, and olfactory dysfunction [3]. These non-motor signs and symptoms are effects of deficits in other neurotransmitters implicated by different brain regions, such as the olfactory bulb, basal ganglia, and frontal cortex, and can occur before the appearance of motor symptoms [3]. Pathologically, the presence of neuronal inclusions known as Lewy bodies in many dopaminergic cells of the SNpc has been seen in many post-mortem findings in patients with PD.

Due to the pathogenesis of PD being multifactorial and the fact that the cause of PD remains unclear, the currently available treatments include pharmacological therapy such as using medications to increase dopamine levels in the brain, deep brain stimulation, and physiotherapy [4]. Although these treatments can reduce the motor symptoms and improve the quality of life of PD patients, there is an urgent need for new therapeutic strategies to prevent, slow, or halt the progression of the disease. Current diagnostic modalities of PD are circumscribed by the fact that there are no specific tests to diagnose PD other than identifying the motor symptoms that the PD patients developed [5]. However, it is estimated that motor symptoms begin to appear when > 30% of dopaminergic neurons have been degenerated [6]. Thus, early diagnosis of PD was always thought to have crucial implications for disease-modifying strategies. For the development of such strategies, the use of appropriate in vitro and in vivo models becomes inevitably valuable to obtain a greater insight into its cause and pathogenesis of PD, in addition to reproducing all clinical and pathological characteristics of PD.

Current in vitro and in vivo PD models can be broadly categorized into genetic and neurotoxin models. Genetic models are used by manipulating genes that have been causally linked to the development of familial PD. However, the neuropathological and behavioral changes evocative to human PD cannot be fully recapitulated in this sophisticated model [7]. On the other hand, neurotoxin models are the most widely used classical PD model, due to their low cost, easy handling, and rapid development in the progression of PD [7]. Various neurotoxin-based models exhibiting degeneration of dopaminergic cells in the SNpc and inducing PD-like phenotypes have been reported by using 6-hydroxydopamine (6-OHDA) and 1-methyl-4-phenyl-1,2,3,6-tetrahydropyridine (MPTP), in addition to other herbicides, such as maneb, rotenone, and paraquat. The use of pesticides and herbicides to model PD has become increasingly important in recent years with the goal of developing neuroprotective agents to halt the progression of PD. This is because exposure to pesticides and herbicides by living in rural areas, farming, or well-water consumption has been implicated with increased risk and incidence for the development of PD [8, 9].

Paraquat is an important member of the bipyridylium family of broad-spectrum, a nonselective fast-acting herbicide that disrupts the intracellular electron transfer system in plants, resulting in the disruption of the plant organelles and ultimately leading to cell death [10]. It is widely used worldwide in many agricultural and non-agricultural settings to control broad-leaved weeds and grasses in many crops, such as cotton, soybeans, sugar cane, and corn. Paraquat has been reported to cause acute poisoning and death due to its toxicity. The common exposure routes of paraquat that would lead to poisoning, either accidentally or intentionally, are ingestion, skin exposure, and inhalation. Paraquat has been used in experimental studies focusing on its pathological effects on the brain, heart, lungs, kidneys, liver, and muscle due to the systemic toxicity and fatality after acute exposure. The interest in using paraquat as a neurotoxin to model PD started since its discovery due to its similarity in terms of its molecular structure and biochemistry with 1-methyl-4-phenylpyridinium (MPP^+^), the active metabolite of MPTP, a neurotoxin that can induce PD-like features in animal models and humans [11]. For many years, studies have demonstrated that individuals exposed to paraquat had a higher risk of developing PD [12–14]. In this article, we will collate evidence of paraquat exposure in relation to PD and discuss paraquat-induced alterations at both cellular and molecular levels. We will first conceptualize paraquat-induced alterations around different pathogenic mechanisms of PD, which can potentially lead to the activation of the apoptotic cell death machinery.

## Paraquat-Induced α-Synuclein Pathology

Neuronal inclusions known as Lewy bodies are present in many dopaminergic cells of the SNpc in many post-mortem findings in patients with PD [15]. The major component of a Lewy body consists of an aggregated intracytoplasmic protein known as α-synuclein [16]. α-Synuclein is an intrinsically disordered and highly dynamic protein that can exist in either soluble monomers or an α-helical multimeric conformation [17]. However, α-synuclein may have the ability to convert from the monomeric form to different oligomeric and aggregated configurations, including spherical and fibrils that are built by recruiting additional α-synuclein monomeric subunits [18]. α-Synuclein is expressed abundantly throughout the brain with a higher concentration in the SNpc [19]. The exact function of α-synuclein is still inconclusive. However, the protein might be involved in synaptic plasticity and neurotransmitter release since it is mainly located in the presynaptic terminal of neurons [20]. At the cellular level, α-synuclein is expressed primarily in the presynaptic terminal of neurons, mitochondria, endoplasmic reticulum (ER), Golgi apparatus, and in the endo-lysosomal system [21]. The exact physiological function of α-synuclein at each subcellular compartment is still poorly understood. However, α-synuclein has been demonstrated to interact strongly with synphilin-1, an adaptor molecule that anchors α-synuclein to cytoplasmic proteins involved in vesicular transport and cytoskeletal function [22].

Cumulative studies suggested the accumulation and aggregation of α-synuclein affect the functional integrity of neurons and can contribute to neurotoxicity [23]. This can be seen in a study by Powers et al. [19], where overexpression of α-synuclein in N27 dopaminergic cells potentiated the paraquat-induced toxicity and metabolic dysfunction compared to normal cells. The role of α-synuclein in contributing to cell vulnerability arises from the misfolding and accumulation of the protein into a membrane-bound pore-like structure resulting in membrane leakage and altered intracellular ionic balance [24]. In addition, α-synuclein interacts with complex I of the electron transport chain (ETC), resulting in higher production of reactive oxygen species (ROS), which in turn alters the expression of mitochondrial genome-encoded genes and induces mitochondrial fragmentation [25].

In recent years, a great deal of evidence has suggested the interaction of α-synuclein with environmental toxicants such as paraquat, resulting in the increased α-synuclein propensity to oligomerize and accumulate. In vitro studies using paraquat were reported to induce a conformational change in α-synuclein and significantly accelerated the α-synuclein fibrillation rate in a dose-dependent manner [26, 27]. In addition, Chorfa et al. [28] reported that paraquat increased the intracellular concentration of α-synuclein in SH-SY5Y cells when compared to other pesticides, such as maneb and rotenone. However, only α-synuclein monomers, as opposed to high molecular mass oligomers or fibrils, were detected [28]. Nevertheless, the protein expression of α-synuclein was upregulated (~ 1.5-fold increase) in the mouse frontal cortex and ventral mesencephalon after 48 h of paraquat (10 mg/kg, i.p., once weekly for 3 weeks) post-treatment; however, the expression returned to baseline levels within a week [27]. A 2.1-fold increase in the α-synuclein protein expression level in the striata was also observed in mice treated with paraquat (10 mg/kg, i.p., twice weekly for 4 weeks) [29]. Fernagut et al. [30] demonstrated the administration of paraquat (10 mg/kg, i.p., once weekly for 3 weeks) caused a 1.9-fold increase in the number of α-synuclein aggregates in the SNpc of mice overexpressing human α-synuclein. However, the α-synuclein aggregates were not detected in saline- and paraquat-treated wild-type mice [30]. In that study, the histological sections of the SNpc had been pretreated with proteinase K to selectively visualize insoluble α-synuclein aggregates [30]. Nonetheless, the findings suggest that α-synuclein over-expression can act synergistically with paraquat to elevate the aggregation of α-synuclein.

Although the underlying initiating mechanism of α-synuclein oligomerization has not been completely deciphered, studies have indicated that the formation of α-synuclein radicals might be a key mechanism. Paraquat has been demonstrated to form α-synuclein radicals in the mid-brain of mice through two distinct mechanisms; (1) the activation of NADPH oxidase and induced nitric oxide synthase (iNOS) in microglia to produce peroxynitrite (ONOO^−^) and (2) leakage of cytochrome C out from the mitochondria into the cytosol to activate the peroxidase activity [31, 32].

There has been a lot of debate over whether the aggregation of α-synuclein is a key feature that contributes to the dysregulation of various cellular processes and cellular toxicity. Manning-Bog et al. [33] demonstrated that mice overexpressing human wild-type or mutated A53T α-synuclein can resist dopaminergic cell degeneration against paraquat and is attributed to the increased expression of heat-shock-protein 70 (Hsp70). This is also consistent with another study where nigral degeneration was not found in mice expressing human wild-type or A53T α-synuclein administered with paraquat (10 mg/kg, i.p., twice weekly for 3 weeks) when compared to their non-transgenic littermates [34]. Moreover, another study demonstrated that MN9D dopaminergic cells overexpressing human wild-type α-synuclein treated with paraquat alone did not exhibit cytotoxicity or compromised membrane integrity [35]. Thus, more studies are required to establish the role of α-synuclein in paraquat-induced neurodegeneration.

## Paraquat-Induced Oxidative Stress

### Increased Lipid Peroxidation

Oxidative stress plays a crucial role in the progressive deterioration of dopaminergic neurons in PD. Efforts have been made to study the oxidative stress markers present in PD patients compared to healthy controls. Recent advancement in diagnostic testing has provided a new approach to analyze biomarkers that are involved in oxidative stress in the blood and cerebrospinal fluid (CSF). Oxidative stress can trigger lipid peroxidation, which can damage cellular membranes, lipoproteins, and other molecules that contain lipids. The brain is particularly susceptible to lipid peroxidation due to its high unsaturated fatty acid levels. A meta-analysis reported by Wei et al. [36] concluded that malondialdehyde (MDA), an end product of lipid peroxidation, was increased in the blood of PD patients. The presence of other end products of lipid peroxidation, such as 4-hydroxynonenal (HNE) and Nε-(carboxymethyl)lysine, were also found in the Lewy bodies of post-mortem PD brain tissues [37]. In addition, it has been shown that MDA levels were doubled in SK-N-SH cells treated with paraquat (14 μM, 24 h) [38]. MDA levels were also increased in the brain of paraquat-treated Drosophila flies [39, 40]. Since MDA is a well-known biomarker of lipid peroxidation [41], the results above suggest that paraquat may induce oxidative stress leading to increased lipid peroxidation in the brain.

### Increased Intracellular ROS Levels

Oxidative stress defines a disequilibrium between the generation and accumulation of ROS in the cells and tissues, and the ability of the biological system to detoxify them through the production of antioxidants. Enhanced ROS production has been implicated in the development of many pathologies, including neurodegenerative diseases such as PD [42]. Accumulating evidence has shown that toxins such as paraquat have been linked to increased oxidative stress. For instance, Alural et al. [43] demonstrated a 1.5-fold increase in ROS levels in SH-SY5Y cells treated with paraquat (500 μM, 24 h). In accordance, Ravi et al. [38] showed a 2-fold increase in ROS generation in SK-N-SH cells treated with paraquat (14 μM, 24 h). Niso-Santano et al. [44] also reported a dramatic increase in the superoxide anion (O_2_^•−^) radicals when SH-SY5Y cells were treated with paraquat (100 µM, 4 h). An in vivo study using Drosophila flies treated with paraquat (20 mM, p.o., 24 h) indicated a 3.7-fold increase of O_2_^•−^ radical in the brain [39]. ROS such as hydrogen peroxide was also doubled in the brain of Drosophila flies when treated with paraquat (20 mM, p.o., 48 h) [40]. The data show that paraquat causes neurodegeneration by increasing intracellular ROS levels.

### Impaired Antioxidant Defense

Glutathione (GSH) is a natural antioxidant found in the body, which plays a significant role in protecting the cells against ROS and reactive nitrogen species (RNS) [45]. A decrease in GSH level in the SNpc of the post-mortem brain of PD patients has been documented [46–50]. GSH level in the blood was also lower in patients with PD [36]. Nevertheless, the deficiency in GSH has been reported to impair the cellular antioxidant defense mechanism. Re-exposure to paraquat (5 mg/kg, i.p., twice weekly for 12 weeks) during adulthood caused a 45% decrease of GSH content in the nigrostriatal tissue of rats that had been previously exposed to paraquat, postnatally [51]. Depletion of GSH in the brain of PD patients may be due to the decrease in the synthesis of GSH. It would be foreseen that the activity of glutamate-cysteine ligase (GCL), the rate-limiting enzyme in GSH synthesis [52], decreases in the brain of PD patients if the alteration in the synthesis of GSH was the cause of GSH depletion. Indeed, the activity of GCL was found to be lower throughout the brain as a result of the aging process [53]. Liang et al. [54] reported a 60% reduction in GSH level in the striatum of GCL knockout mice. Lam, Ko [55] reported a 30% reduction in the GCL activity in differentiated PC12 cells treated with paraquat (150 μM, 24 h). GSH depletion may be contributed by increased efflux of glutathione disulfide (GSSG) mainly out of glial cells [56]. Intracellular glutathione levels are maintained at a redox equilibrium between GSSG and GSH [57]. However, oxidative stress will occur if the equilibrium is disrupted and altered toward GSSG [58]. This was seen in a study by Djukic et al. [59], where the authors reported a significantly higher GSSG and GSSG/GSH ratio in the bilateral cortex of adult Wistar rats treated with paraquat (2.5 μg/10 μL, 24 h), intrastriatally.

Other antioxidant enzymes, such as catalase (CAT), superoxide dismutase (SOD), and glutathione peroxidase (GPx), play a prominent role in antioxidant defense [60]. Kish et al. [61] concluded a slight but significant decrease in GPx activity in several post-mortem brain regions, including SNpc of PD patients. The results were consistent with other animal models resembling PD, such as in Tang et al. [62], where the authors signified a reduction in GPx activity in the midbrain of Sprague–Dawley rats treated with paraquat (10 mg/kg, i.p., once weekly for 4 weeks). Similar to the peroxidase activity, the activity of CAT was also decreased in the SNpc of PD patients [63]. CAT level in the blood was also reduced in patients with PD [36]. Shukla et al. [39] reported that paraquat (20 mM, p.o., 24 h) induced a significant decrease in SOD activity by ~ 50% in the brain of the Drosophila flies. These findings indicate that paraquat causes oxidative damage by lowering the antioxidant defense.

In contrast, Srivastav et al. [40] showed a 2.2-fold upregulation in SOD activity in the head of the Drosophila flies when the flies were treated with paraquat (20 mM, p.o., 48 h). CAT activity in the brain of paraquat-treated flies was also higher (~ 1.6-fold) [40]. The increase in SOD and CAT activities may be a protective response to elevated levels of free radicals in paraquat-treated flies [39, 40]. In accordance, several studies showed that SOD activity was also higher in the SNpc and erythrocytes of PD patients [64–66].

### Downregulated Nrf2-Keap1-ARE Signaling Pathway

Nuclear factor erythroid 2-related factor 2 (Nrf2) is an essential transcription factor that regulates a wide range of antioxidant defense pathways, leading to the production of various antioxidant enzymes [67]. Under normal conditions, the abundance of intracellular Nrf2 present in the cytoplasm is consistently low due to the rapid degradation of Nrf2 via the ubiquitin-proteasomal pathway [68]. The degradation of Nrf2 occurs when Nrf2 is bound to Kelch-like ECH-associated protein 1 (Keap1) located in the cytoplasm [68]. In response to oxidative stress, Keap1-mediated degradation of Nrf2 is inhibited, resulting in the translocation and accumulation of Nrf2 in the nucleus [69]. Nrf2 then binds to antioxidant response element (ARE), activating many antioxidant and cytoprotective genes, such as GSH, SOD, CAT, glutathione-*S*-transferase (GST), NAD(P)H dehydrogenase (quinone) 1 (NQO1), and heme oxygenase-1 (HO-1) [68]. Petrillo et al. [70] demonstrated a significant increase in Nrf2 mRNA and protein expression in leukocytes of PD patients compared to healthy individuals. In autopsy brain tissue obtained from PD patients, nuclear translocation of Nrf2 was found to be more abundant in the dopaminergic neurons in the SNpc, but this response may not be sufficient to protect neurons from cell death [71]. An in vivo study using Drosophila flies showed that treatment with paraquat (20 mM, p.o., 48 h) resulted in the upregulation of Nrf2 mRNA expression by 1.6-fold [40]. Also, HO-1 cDNA expression by immunoblotting was found to be upregulated (~ 4-fold higher) in SH4741 dopaminergic neuronal cell line treated with paraquat (800 μM, 20 h) [72]. Paraquat (500 μM, 24 h) caused more cell death in Nrf2 knockdown SH-SY5Y cells than control cells [43], suggesting that Nrf2 plays an essential role in neuroprotection. Alural et al. [43] reported a reduction in the expression of NQO1 and HO-1 mRNAs in Nrf2 knockdown SH-SY5Y cells. These studies indicated that paraquat-induced oxidative stress could trigger the Nrf2-Keap1-ARE pathway to initiate the downstream antioxidant responsive elements, including NQO1 and HO-1 mRNA expression, to prevent oxidative injury.

## Paraquat-Induced Nitrosative Stress

More evidence has suggested that RNS is involved in mediating nitrosative stress. RNS is generated by the rapid reaction between O_2_^•−^ radicals and nitric oxide, which results in the production of ONOO^−^ [73]. The instability of ONOO^−^ stabilizes itself by donating the -NO_2_ functional group to Tyr residues of proteins that form the neuronal cytoskeleton, resulting in the formation of 3-nitrotyrosine [74]. This results in structural alteration of proteins, which ultimately leads to the death of dopaminergic neurons [75]. 3-Nitrotyrosine has been well known as a potential biomarker of oxidative and nitrosative stress associated with numerous pathological conditions and disorders of the central nervous system, including PD [74]. Fernández et al. [76] reported elevated levels of free 3-nitrotyrosine and nitroalbumin in the serum and CSF of patients with early PD. In addition, the authors reported the presence of nitro-α-synuclein in serum but not in CSF. Paraquat (10 mg/kg, s.c., twice weekly for 3 weeks) induced a higher magnitude of increase in 3-nitrotyrosine level in GCL knockout mice compared to their wild-type counterpart [54]. In addition, paraquat (20 mM, p.o., 24 h) has also been shown to cause a 2.6-fold increase in the concentration of ONOO- in the brain of Drosophila flies [39]. Thus, oxidative and nitrosative stress plays a crucial role in paraquat-induced neurodegeneration.

## Paraquat-Induced Impairment of Dopamine Catabolism

Several lines of evidence have suggested that biochemical defect in dopamine catabolism is implicated in patients with PD. The concentration of dopamine in the SNpc is strictly regulated. Increased dopamine levels, dopamine oxidation, and its reactive catabolites have been suggested as the major oxidative stressor resulting in neuronal death in PD [77]. Dopamine catabolism starts a reaction known as oxidative deamination catalyzed by monoamine oxidase, producing hydrogen peroxide, ammonia, and 3,4-dihydroxyphenylacetaldehyde (DOPAL) [78]. DOPAL is further metabolized to 3,4-dihydroxyphenylacetic acid (DOPAC) or 3,4-dihydroxyphenylethanol (DOPET) by aldehyde dehydrogenase [78]. A post-mortem examination on the brain of sporadic PD patients revealed a decrease in dopamine, DOPAL, and DOPAC levels in the putamen, caudate, and cortex [79]. Other studies also reported elevated DOPAL:DOPAC ratio, in addition to decreased aldehyde dehydrogenase activity in the putamen [80]. Furthermore, a reduced level of DOPAC was also seen in the CSF of PD patients [81]. Although DOPAL is a physiological intermediate in dopamine catabolism, it can also act as a potent neurotoxin. DOPAL injection into the SNpc resulted in a more prominent loss of dopaminergic neurons when compared to dopamine, DOPAC, or DOPET [82].

Apoptosis of the dopaminergic neurons is associated with reducing dopamine and increasing DOPAC levels [83]. Drosophila flies treated with paraquat (20 mM, p.o., 24 h) showed a decrease in dopamine level by 63% and a 2.9-fold increase in DOPAC level in the brain [39]. In addition, a concentration-dependent decrease in the number of dopaminergic neurons was seen at 24 h and 48 h. Tyrosine hydroxylase (TH) is a rate-limiting enzyme in the synthesis of dopamine and TH expression is decreased when the dopaminergic neuronal loss occurred [77]. Exposure to paraquat, for example, can generate ONOO^−^, which leads to the nitration of the tyrosine residue in TH, resulting in the loss of its enzymatic activity and subsequently permanent loss of TH-positive neurons [84, 85]. Dwyer et al. [86] showed that the loss of TH-positive neurons persisted throughout the 6 months after the last paraquat injection (10 mg/kg, i.p., every other day for 2 weeks) on C57Bl6 mice. The data above suggest that paraquat-induced dopaminergic cell death may be linked to the alteration of dopamine catabolism.

## Paraquat-Induced Reduction of Brain-Derived Neurotrophic Factor

Among the proteins putatively involved in the pathogenesis of PD, neurotrophic factors play an important role in the differentiation, maturation, maintenance, and survival of mammalian neurons [87]. These factors have also been shown to have neurogenic and neuroprotective effects under adverse conditions, such as cerebral ischemia [88], neurotoxicity [89], glutamatergic stimulation [90], and neurodegenerative diseases [90]. One of the well-studied neurotrophic factors is brain-derived neurotrophic factor (BDNF), a member of the neurotrophin family, identified in most brain regions, including the ventral midbrain, ventral tegmental area, and SNpc [91]. The biosynthesis of BDNF involves the precursor protein pre-pro-BDNF in the ER to be processed to pro-BDNF upon the cleavage of the signal peptide [92]. Pro-BDNF is transported to the Golgi apparatus to be packaged into secretory vesicles and is released from the neurons, either constitutively or in an activity-dependent manner. It may also be converted into mature BDNF by members of the subtilisin/kexin family of endoproteases such as furin, or by the action of plasmin through tissue plasminogen activator (tPA) catalysis [93]. Mature BDNF has been known to augment neuronal synaptogenesis and dendritogenesis and to improve synaptic plasticity upon binding with TrkB receptors, resulting in neuronal development and survival [94]. On the contrary, pro-BDNF acts through the p75 neurotrophin receptor (p75^NTR^), and the activation of the receptor can oppose those effects elicited by the mature BDNF/TrkB signaling, such as cell death, retraction of the neuronal growth cone, and pruning of axonal processes [95]. For many years, evidence has suggested the importance of BDNF as one of the critical factors in establishing the proper number and function of dopaminergic neurons in the SNpc [96].

Downregulation of BDNF mRNA and protein specifically in the SNpc of patients with PD might participate in the death of the nigral dopaminergic neurons [97–99]. Alural et al. [43] demonstrated that BDNF mRNA expression and secreted protein levels were decreased by > 60% in SH-SY5Y cells treated with paraquat (500 μM, 24 h). The study also showed a decrease in the neurite number and length by 50% and 15%, respectively, which indicates the importance of BDNF in promoting neuron survival and growth [43]. Moreover, paraquat has been reported to downregulate BDNF protein expression in the hippocampus [100, 101] and mRNA expression in the striatum of C57BL/6 male mice after repeated administration for a total duration of 3 weeks [102]. Regardless, the action of paraquat on pro-BDNF and mature BDNF was not specified from the studies above. Moyano et al. [103], however, reported a concentration-dependent decrease in the protein concentration of both pro-BDNF and mature BDNF, in addition to TrkB and tPA in primary hippocampal cells treated with paraquat. Moreover, upregulation of the protein expression of p75NTR can be observed in the study [103].

Nonetheless, there are conflicting reports on the circulating level of BDNF in patients with PD [98, 99]. The discrepancy could be due to the vast clinical heterogeneity in PD, particularly the variability in disease severity, subtypes, and duration. Moreover, many factors, including gender, exposure to various medications, and individual cognitive performances, have been documented to affect circulating BDNF levels in PD patients [100–102]. In accordance, hippocampal BDNF protein expression was increased by > 65% in female C57BL/6 mice treated with paraquat (10 mg/kg, i.p., 3 times a week for 3 consecutive weeks) [104]. However, there was no significant difference in the protein expression of CREB, the primary mediator of BNDF transcriptional regulation [104]. Thus, it is tempting to speculate that sexual dimorphism may play a role in paraquat-associated BDNF expression occurring in the hippocampus and potentially in other areas of the mouse brain. The female sex hormone estrogen has been well documented to upregulate transcription of BDNF by interacting with the estrogen response element in the BDNF gene [105]. The mRNA expression of BNDF was upregulated at 48 h and 72 h after exposure of U118 astroglia to paraquat (250 μM) [106, 107]. However, paraquat also upregulates the expression of other pro-inflammatory astrocytic factors such as interleukin (IL)-1β and IL-6 resulting in cell cycle arrest, indicating the increase in the expression of neurotrophic factors could be a compensatory mechanism to neuronal insult [106]. Moreover, upregulation of BDNF as a compensatory mechanism against neuronal damage has also been reported in other neurodegenerative diseases. In Alzheimer’s disease, for instance, the compensatory increase of BDNF occurs at the early stage and is then followed by a drop as the disease progresses to the advanced stage [108].

## Paraquat-Induced ER Stress

The ER is the largest membrane-closed cellular organelle in all eukaryotes, which plays a crucial role in the synthesis of protein and lipid, as well as functioning as a free calcium reservoir [109]. Initial protein maturation steps occur in the ER where the synthesized proteins in the secretory pathways are precisely folded. However, under certain circumstances, physiological stresses such as glucose starvation, hypoxia, oxidative stress, and disruption of calcium homeostasis can disrupt the protein folding at the ER, leading to the accumulation of unfolded and misfolded proteins, a cellular condition termed as ER stress [110]. The accumulation of misfolded protein in the ER that cannot be effectively removed by the protein degradation mechanism such as ubiquitin–proteasome system can ultimately result in neuronal death [111]. Paraquat has been demonstrated to impair the activity of the proteasomal system, which is a late event in the progression of cell death [112, 113].

Upon ER stress, cells can activate a cascade of mechanisms to cope with protein folding alterations, which is termed the unfolded protein response (UPR). Activation of the UPR transduce information about the status of protein folding in the ER lumen to other organelles, such as the nucleus and cytosol, to mitigate any further accumulation of unfolded protein load [114]. The consequences of activating the UPR are the enervation in the rate of protein synthesis, upregulation of genes encoding chaperones and other proteins involved in the protein degradation or prevention of protein aggregation, participation of protein folding and stabilization, and lastly, translocation of proteins to other cellular compartments [115]. The UPR is mainly controlled by three major transmembrane stress sensors, namely protein kinase RNA-like endoplasmic reticulum kinase (PERK) or eIF2α kinase, activating transcriptional factor 6 (ATF6), and inositol-requiring transmembrane kinase/endoribonuclease 1 (IRE1), which orientate with their luminal domain in the ER lumen and their signal transduction domain toward their cytoplasm [116].

Under physiological conditions, an ER-resident chaperone known as glucose-regulated protein of 78 kDa (GRP78) binds to the luminal domains of PERK, which keeps the kinase in an inactive state [117]. However, when unfolded and misfolded proteins accumulate in the ER, GRP78 dissociates from PERK and allows the dimerization and autophosphorylation of PERK, thus activating it [117]. The activation of PERK phosphorylates the α subunit of the eIF2α, consequently resulting in the induction of signal transduction events that activate downstream UPR target genes, such as GRP78 and activating transcriptional factor 4 (ATF4) [110]. On the contrary, the ATF6 sensor protein is triggered due to the accumulation of misfolded proteins and UPR activation. This results in the translocation of the protein to the Golgi apparatus, where it undergoes proteolysis producing a free cytosolic domain that triggers transcriptional regulation of ER chaperone proteins, such as GRP78 and growth arrest- and DNA damage-inducible gene 153 (GADD153)/C/EBP-homologous protein (CHOP) [118, 119]. Nonetheless, IRE1 also monitors ER homeostasis and activates its intrinsic RNase activity upon ER stress through conformational change, homodimerization, and autophosphorylation [120]. The activation of the RNAse activity of IRE1 results in the generation of an active spliced isoform of the transcription factor, X-box-binding protein 1 (XBP-1) [121]. Subsequently, XBP-1 translocates to the nucleus and modulates gene expression of molecular chaperones and proteins, attributing to ER-associated degradation by binding to the promoters of its target gene [122].

In human PD post-mortem brain tissue, the expression of UPR activation markers, such as p-PERK and p-eIF2α, were upregulated in the dopaminergic neurons of the SNpc [123, 124]. In another study, Baek et al. [125] showed an upregulation of the GRP78 and p-PERK protein levels in the cingulate gyrus of PD patients. More recently, Baek et al. [126] concluded a significant upregulation of GRP78 mRNA level in all brain regions in PD patients compared to control subjects. Nonetheless, the eIF2α mRNA level was not significantly different in any of the brain regions compared to the control group [126]. However, in a study by Esteves, Cardoso [127], the protein expression levels of GRP78 and ATF4 were found to be downregulated in the SNpc of PD-post-mortem brain samples. Interestingly, a study reported colocalization of p-IRE1 with α-synuclein in the SNpc of PD patients, indicating that the accumulation of α-synuclein contributes to the activation of IRE1/XBP-1 of the UPR [128]. In line with these studies, a time-dependent increase in the protein expression of GRP78, glucose-regulated protein of 78 kDa (GRP94), and p-eIF2α in the dopaminergic N27 cells treated with paraquat (500 μM, 12–48 h) was also observed [129]. Moreover, protein expression of p-PERK, p-eIF2α, ATF6, p-IRE1, XBP-1, and immunoglobulin heavy chain binding protein (BiP) was found to be upregulated in adrenal pheochromocytoma PC12 cells treated with paraquat (1 mM, 24 h) [130], suggesting that paraquat plays an important role in causing ER stress.

Physiological processes requiring a high demand for protein synthesis can activate the UPR without triggering the apoptotic pathway [131]. However, conditions that lead to prolonged ER stress can often cause cellular dysfunction and cell death. Chronically sustained activation of eIF2α upregulates the pro-apoptotic transcription factor GADD153/CHOP [132]. Upregulation of both the mRNA and protein expression of GRP78 and CHOP was observed in the SNpc region of PD post-mortem brains compared to the age-matched control group [133]. However, another study by Baek et al. [126] reported that CHOP mRNA levels in all brain regions were not significantly different between PD patients and control subjects. Paraquat has been demonstrated to increase the mRNA expression of CHOP in a concentration-dependent manner in SH-SY5Y cells [134]. Overexpression of CHOP has been reported to result in cell cycle arrest and ER stress-induced apoptosis [135]. This was demonstrated in two independent studies by Chinta et al. [129] and Huang et al. [130], where an upregulation of protein expression of CHOP and other apoptotic markers, such as caspase-3, caspase-7, and cleaved poly(ADP-ribose) polymerase (PARP) in vitro, indicating the activation of ER stress-induced apoptosis.

## Paraquat-Induced Mitochondrial Dysfunction

Many lines of evidence suggested that impairment of the mitochondrial function has been linked to the pathogenesis of PD [136]. An increased level of deleted mitochondrial DNA was observed in the SNpc of PD patients, suggesting respiratory chain deficiency and mitochondrial dysfunction [137]. A reduction in the metabolic activity and protein level of NADH dehydrogenase or mitochondrial complex I in the SNpc and frontal cortex of post-mortem examination in PD patients has been reported [138, 139]. In addition, a recent study has shown the deficiency in the mitochondrial complex I throughout the brain of PD patients [140]. Furthermore, brain mitochondria from PD patients showed functional impairment and mis-assembly of complex I [141]. Mitochondrial complex I is the first enzyme present in the mitochondrial ETC, which functions to translocate protons from the mitochondrial matrix to the intermembrane space, generating an electrochemical gradient to produce ATP. The disruption of mitochondrial complex I activity leads to the inefficient generation of ATP and an increased level of ROS in the neurons. Therefore, complex I is considered to be an important site of ROS generation. Nonetheless, the causes and consequences of mitochondrial complex I deficiency in SNpc neuronal cells remain to be explored in the future. Regardless, Choi et al. [142] demonstrated that dopaminergic neurons from NADH: ubiquinone oxidoreductase subunit S4 (NDUFS4) knockout mice that are complex I-deficient appeared normal and healthy with no decrease in survival when compared to neurons from wild-type mice.

The direct linkage of mitochondrial dysfunction with PD came from neurotoxins such as paraquat. A study conducted by Fukushima et al. [143] demonstrated a decrease in mitochondrial complex I activity and an increase in lipid peroxidation in the brain of bovine treated with 500 μM paraquat. Mitochondrial complex I transfers two electrons from NADPH to ubiquinone, resulting in the oxidation of NADPH to NADP^+^. Upon entry into cells, paraquat dication (PQ^2+^) undergoes redox cycling where it disrupts the oxidation of NADPH by accepting electrons to form paraquat mono-cation radical (PQ^•+^) through NADPH-cytochrome P450 reductase, ultimately inhibiting mitochondrial complex I activity [144, 145]. In a study by Srivastav et al. [40], ATP levels were decreased in the Drosophila flies when treated with paraquat (20 mM, p.o., 48 h). Choi et al. [142] have suggested that the inhibition of mitochondrial complex I is not a pivotal factor to paraquat-induced dopaminergic neuronal death. In that study, the dopaminergic neurons from NDUFS4 knockout mice did not show increased sensitivity to paraquat (50 μM, 24 h) or MPP^+^ [142]. However, another PD-mimicking neurotoxin, rotenone, showed increased TH-positive neuronal loss in NDUFS4 knockout mice compared to wild-type mice [142]. Nonetheless, the potential for paraquat to induce mitochondrial dysfunction on dopaminergic neurons warranted further investigation.

The mitochondrial membrane potential (ΔΨm) generated by the proton pumps of the ETC, i.e., complex I, complex III, and complex IV, plays a crucial role in storing energy in the form of ATP during oxidative phosphorylation. Under basal conditions, cells maintain a stable intracellular ΔΨm, which is essential for maintaining normal cellular homeostasis [146]. Alteration to the ΔΨm can have deleterious effects on cells. Paraquat has been shown to impair the ΔΨm, as evidenced in a study by Kang et al. [147], where a decrease in the ΔΨm in PC12 cells by 75% was seen when the cells were treated with paraquat (300 μM, 24 h). The loss of ΔΨm has been widely due to the opening of the mitochondrial permeability transition pore, a transmembrane protein residing in the inner mitochondrial membrane [148]. Once the ΔΨm has collapsed, the cells are committed to the apoptotic pathway due to energy depletion [149].

Cytochrome c, a protein located in the inner mitochondrial membrane, plays a vital role in shuttling electrons from complex III and complex IV in the ETC [150]. Cytochrome c is undoubtedly an essential player in the apoptotic pathway of cells. Although it is a ‘quiet worker’ in the ETC, the induction of apoptotic stimuli causes cytochrome c to be released from the mitochondria to the cytosol to initiate the recruitment of caspase-9 and maturation of other caspases that eventually mediates the biochemical and morphological features of apoptosis [150]. Studies have shown that paraquat can induce cytochrome c release from the mitochondria to the cytosol of neuroblastoma cell lines in a concentration-dependent fashion [44, 151]. In addition, Yang et al. [152] co-transfected cells with a vector designed for fluorescent labeling of mitochondria and another vector for the fluorescent labeling of pro-apoptotic proteins. SH-SY5Y cells treated with paraquat were then fixed and subjected to confocal microscopy. Besides cytochrome c, Yang et al. [152] also reported that other mitochondrial pro-apoptotic proteins, such as DIABLO and HTRA2, were also released upon paraquat exposure (300 μM, 24 h). DIABLO and cytochrome c are released from the intermembrane space of the mitochondria into the cytosol [153]. While cytochrome c directly activates Apaf-1 and caspase-9, DIABLO interacts and removes multiple inhibitor of apoptosis proteins (IAPs) that inhibit both initiator and effector caspases [153]. Thus, removing IAPs by DIABLO frees up the caspases and consequently activates the apoptotic mechanism.

## Paraquat-Induced Apoptosis

Dopaminergic neuronal cell death in the SNpc is a defining feature of PD [154]. Apoptosis or programmed cell death is thought to be the primary mechanism contributing to dopaminergic neuronal cell death [155]. Neuronal apoptosis is an evolutionarily well-conserved process where it plays a vital role in many physiological processes, particularly in the development and maturation of the nervous system [156]. Several studies have suggested that paraquat can induce apoptosis in the dopaminergic neuronal cells in vitro and in vivo, ultimately resulting in cell death. Flow cytometric assessment by Ju et al. [157] using Annexin V-FITC dye reported a 3.8-fold increase in the total percentages of early and apoptotic SH-SY5Y cells when treated with paraquat (300 μM, 24 h). Another study by Alural et al. [43] showed that paraquat (500 μM, 24 h) caused a 1.8-fold increase in the percentage of SH-SY5Y apoptotic cells in the sub G1 phase of the cell cycle.

Apoptosis is characterized by a series of specific morphological events in which the onset begins with the cell and nuclear shrinkage in addition to condensation of the chromatin in the nucleus [158]. Extensive plasma membrane blebbing also occurs during this stage. Later on, the nucleus of the cells progressively condenses and fragments [158]. Apoptotic characteristics, such as nuclear condensation, chromatin fragmentation, and apoptotic chromatic changes, were reported in the SNpc dopaminergic neurons of PD patients [159]. Chen et al. [160] demonstrated that paraquat (400 μM, 24 h) significantly increased the number of N27 dopaminergic neuronal cells with fragmented apoptotic nuclei and multiple chromatin condensation. Nonetheless, one of the hallmarks of apoptosis is internucleosomal DNA fragmentation, which has been demonstrated together with the typical morphological events described above [158]. DNA fragmentation occurs when endogenous DNases excise the internucleosomal region into double-stranded DNA fragments of 180–200 bps [161]. This was confirmed by Chun et al. [72] in which the nuclear DNA of dopaminergic neuronal cells was isolated and separated on an agarose gel and found out that paraquat (800 μM, 12 h) resulted in a significant number of cells undergoing DNA fragmentation as indicated by the presence of multiple bands on the gel. Another method of detecting DNA fragmentation is known as the TUNEL assay. A study showed that exposure of PC12 cells to paraquat (300 μM, 24 h) caused a significant 3.7-fold increase in the number of TUNEL-positive cells, indicating that paraquat induces apoptosis in PC12 cells [147]. Similarly, a lower dose of paraquat (14 µM, 24 h) also caused a 5.5-fold increase in the number of TUNEL-positive SK-N-SH neuroblastoma cells [38].

The initiation of apoptosis is a tightly regulated and controlled process since it is irreversible once activated. To date, research has indicated that there are two main pathways to initiate apoptosis; extrinsic (death receptor pathway) and intrinsic (mitochondrial pathway) [162]. While there is some consensus that the extrinsic pathway contributes to the mechanism of neuronal loss in PD, its role remains unclear; thus, it will not be explored in this review. Regardless, the intrinsic pathway is initiated inside the cells by many endogenous and exogenous stimuli, including ischemia, oxidative stress, and DNA damage. A key player to the intrinsic pathway is the mitochondria, where the outer membrane of the mitochondria membrane becomes permeable and releases apoptogenic proteins such as cytochrome c, which normally exists in the mitochondrial intermembrane space, to the cytosol [163]. This process, which is also known as mitochondrial outer membrane permeabilization (MOMP), is mediated and controlled by the balance between pro-apoptotic (i.e., Bak and Bax) and anti-apoptotic Bcl-2 family proteins (i.e., Bcl-2) [163]. Elevated Bax level has been reported in the SNpc of PD patients [149]. The level of Bcl-2 mRNA expression was reduced in SK-N-SH and SH-SY5Y neuroblastoma cell lines upon exposure to paraquat [151, 164]. Exposure of paraquat (500 μM, 24 h) in SH-SY5Y cells resulted in a 2.6-fold increase in the pro-apoptotic Bax mRNA expression and a 1.7-fold increase in Bax protein expression [43]. Fei et al. [151] demonstrated a higher level of the pro-apoptotic protein Bak in SK-N-SH cells and SNpc of C57BL/6 mice, by 220% and 30%, respectively, when treated with paraquat.

Caspases are widely expressed in most cells as inactive zymogens and activate other procaspases, allowing the protease cascade initiation [165]. The activation of caspases results in the amplification of the apoptotic signaling pathway, leading to rapid programmed cell death. Caspases-8 and -9 are initiator caspases that initiate the entire apoptotic cascade. In contrast, caspase-3, -6, and -7 are executioner caspases that carry out mass proteolysis by degrading cellular components [166]. Furthermore, increased activity and protein expression of caspase-3 was reported in the SNpc of PD patients [167, 168]. In addition, active caspase-8 and caspase-9 were also detected in the SNpc from autopsied PD patients but were not detected in normal controls [169]. Ju et al. [157] reported a 1.9-fold increase in caspase-9 protein expression in SH-SY5Y cells treated with paraquat (300 μM, 24 h). In vitro studies using N27 rat dopaminergic neuronal cells have demonstrated that caspase-3 and -7 protein expression in addition to caspase activity were upregulated in dopaminergic N27 cells upon exposure to paraquat (500 μM, 48 h) [129]. Srivastav et al. [40] reported a 4-fold increase in the mRNA expression and a 3-fold increase in the protein expression of caspase-3 in the head section of Drosophila flies after treatment with paraquat (20 mM, p.o., 48 h).

One of the several vital proteins responsible for cellular functioning and survival is PARP. PARP is a highly conserved and multifunctional nuclear protein that plays a vital role in repairing single- and double-stranded bases in DNA [170]. Activation of PARP-1 catalyzes the transfer of negatively charged ADP-ribose moieties from cellular NAD^+^ to many proteins on specific amino acid residues, generating nicotinamide and ADP-ribose as by-products [170]. Successive addition of ADP-ribose unit to form a long and branched-chain of poly(ADP-ribose), also known as the poly(ADP-ribosylation), forms a scaffold. It then recruits other proteins critical in the DNA repair mechanism. Cleavage of PARP-1 by caspases is considered a prominent hallmark of apoptosis and is responsible for the inactivation of the poly(ADP-ribosylation) process [171]. Cleavage of PARP-1 by caspase-3 has been implicated in several neurological diseases such as PD [171]. A large body of evidence has shown that PARP-1 is cleaved by caspase-3, -7, and -9. Nevertheless, it has also been described that paraquat induces PARP activation in vitro. Chinta et al. [129] reported a 4.8-fold increase in the protein expression of cleaved PARP after paraquat treatment (500 μM, 24 h); however, the protein expression was observed to decrease at 48 h.

## Conclusions

**Fig. 1:**
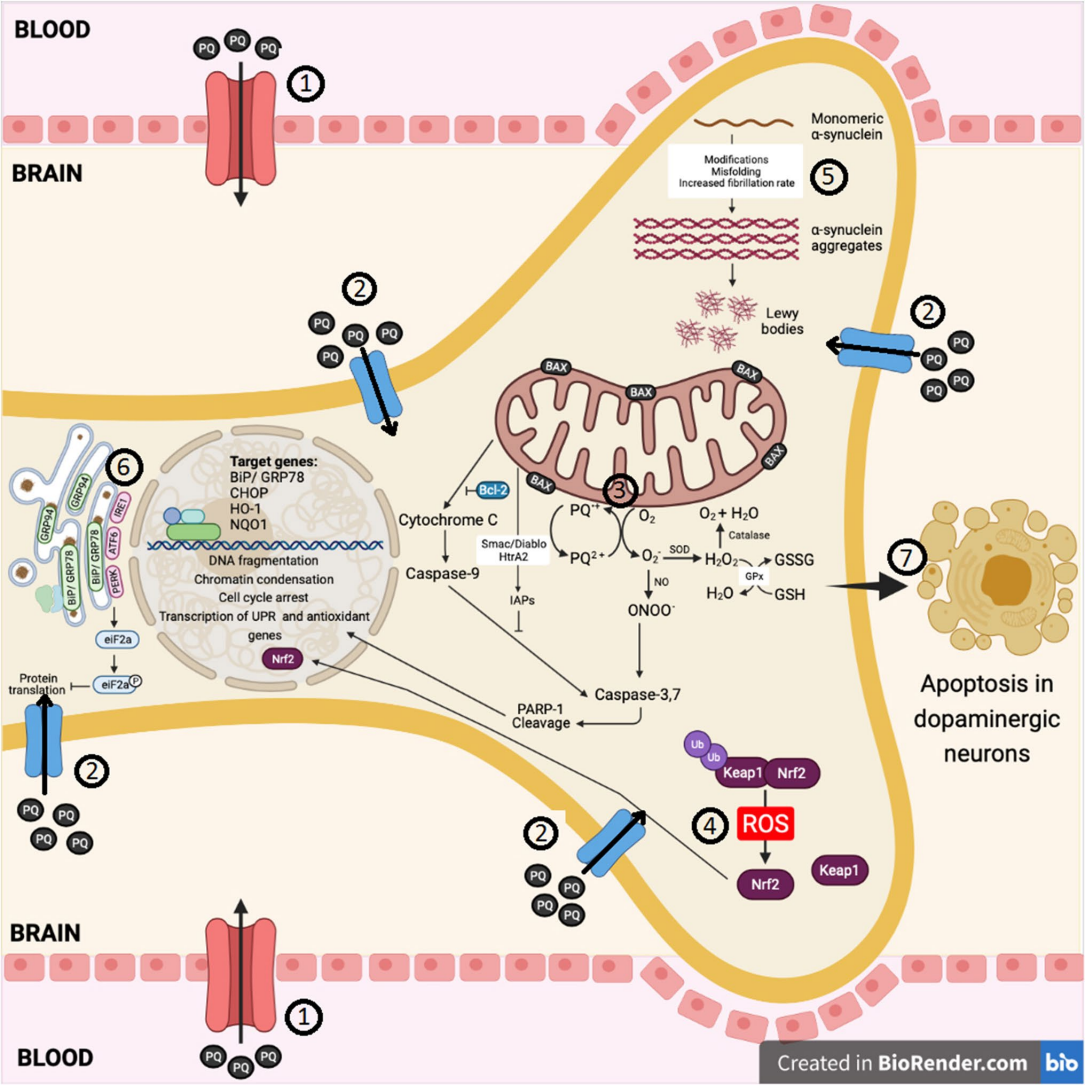
The major molecular targets of paraquat that lead to cellular damage and apoptosis. (1) Paraquat crosses the blood–brain barrier via LAAT. (2) It then enters neuronal cells via transporters such as dopamine transporter DAT, OCT2, and OCT3. (3) Upon entry to the cells, paraquat undergoes a process of redox cycling, a process of alternate reduction and reoxidation. Paraquat is reduced by enzymes present in the mitochondria to form a monocation free radical, PQ^•+^. PQ^•+^ is then rapidly reoxidized in the presence of oxygen to generate O_2_^•−^ and regenerates its parent compound PQ^2+^. If there is sufficient NADPH as an electron donor and O_2_ as an electron acceptor, paraquat will repeatedly undergo the reduction–oxidation cycle, generating O_2_^•−^. This results in the initiation of a reaction cascade leading to ROS generation, such as H_2_O_2_ and OH^−^, in addition to RNS such as ONOO^−^. (4) The generation of ROS and RNS are counterbalanced by the activation of the Nrf2-Keap1-ARE signaling pathway to activate endogenous antioxidant enzyme genes, such as HO-1 and NQO1. (5) Paraquat increase α-synuclein modifications, misfolding, and fibrillation rate resulting in aggregation to form Lewy bodies which are toxic to the cell. (6) Paraquat can also induce ER stress by activating the UPR signal proteins, such as PERK, ATF6, and IRE1, consequently leading to the upregulation of the pro-apoptotic transcription factor, CHOP. (7) Ultimately, the activation of various pathological cellular processes, such as oxidative and nitrosative stress, ER stress, and mitochondrial dysfunction, results in the apoptotic pathway activation, leading to the cell death of dopaminergic neurons. *ARE* antioxidant response element, *ATF6* activating transcription factor 6, *BiP* binding immunoglobulin protein, *CHOP* C/EBP-homologous protein, *DAT* dopamine transporter, *ER* endoplasmic reticulum, *GPx* glutathione peroxidase, *GRP78* glucose regulatory protein 78, *GRP94* glucose regulatory protein 94, *GSH* glutathione, *GSSG* glutathione disulfide, *H*_*2*_*O* water, *H*_*2*_*O*_*2*_ hydrogen peroxide, *HO*-*1* heme oxygenase-1, *HtrA2* HtrA serine peptidase 2, *IAPs* inhibitor of apoptosis proteins, *IRE1* inositol-requiring transmembrane kinase/endoribonuclease 1, *Keap1* Kelch-like ECH-associated protein 1, *LAAT* l-neutral amino acid, *NO* nitric oxide, *NQO1* NAD(P)H dehydrogenase (quinone) 1, *Nrf2* nuclear factor erythroid 2-related factor 2, *O*_*2*_ oxygen, *O*_2_^•−^ superoxide anion, *OCT2* organic cation transporter 2, *OCT3* organic cation transporter 3, *ONOO*^−^ peroxynitrite, *PARP*-1 poly (ADP-ribose) polymerase-1, *PERK* protein kinase RNA-like endoplasmic reticulum kinase, *PQ* paraquat, *PQ*^•+^ paraquat monocation free radical, *PQ*^*2*+^ paraquat dication, *ROS* reactive oxygen species, *Smac*/*DIABLO* second mitochondria-derived activator of caspase/direct inhibitor of apoptosis-binding protein, *SOD* superoxide dismutase, *Ub* ubiquitin, *UPR* unfolded protein response

The mechanisms of PQ-induced toxicity are summarized in Fig. 1. The synthesized review has strongly demonstrated the relationship between paraquat and PD as a strong inducer of oxidative stress, which contributes ROS formation. PD is a multifactorial disease involving many biochemical pathways, such as oxidative injury, mitochondrial dysfunction, ER stress, alteration in dopamine catabolism, inactivation of TH, and decrease in the neurotrophic factor BDNF, ultimately resulting in apoptosis of the dopaminergic neurons in the SNpc. Thus, the use of in vitro and in vivo models using paraquat, which directly or indirectly contributes to the pathogenesis of the disease under the exacerbated condition of oxidative stress, may provide us with a larger picture to develop new therapeutic targets in the near future. The production of ROS and RNS such as O_2_^•−^ and ONOO^−^ via redox cycling of paraquat is widely proposed to be the key mechanism of oxidative stress resulting in the apoptosis of dopaminergic neurons. Like innumerable reviews, we identified consistency among studies that investigate the cellular processes and in vivo organisms, in addition to the human population in the framework of PD pathogenesis. The pathophysiological processes involving laboratory animals and the affected signaling cassettes we reviewed in this paper identifying the development of PD characteristics following exposure to paraquat provides imperative evidence to expand this research and solve this problem.

## Data Availability

Not applicable.
